# Evaluation of the first two Frontline cohorts of the field epidemiology training program in Guinea, West Africa

**DOI:** 10.1186/s12960-022-00729-w

**Published:** 2022-05-12

**Authors:** Doreen Collins, Boubacar Ibrahima Diallo, Mariama Boubacar Bah, Marlyatou Bah, Claire J. Standley, Salomon Corvil, Lise D. Martel, Pia D. M. MacDonald

**Affiliations:** 1 RTI International, Research Triangle Park, NC USA; 2 RTI International, Conakry, Guinea; 3 Center for Global Health Science and Security, Georgetown University, Washington, DC USA; 4 African Field Epidemiology Network, Conakry, Guinea; 5 US Centers for Disease Control and Prevention, Atlanta, GA USA

**Keywords:** Workforce development, Field epidemiology

## Abstract

**Background:**

The 2014–2016 Ebola virus disease outbreak in West Africa revealed weaknesses in the health systems of the three most heavily affected countries, including a shortage of public health professionals at the local level trained in surveillance and outbreak investigation. In response, the Frontline Field Epidemiology Training Program (FETP) was created by CDC in 2015 as a 3-month, accelerated training program in field epidemiology that specifically targets the district level. In Guinea, the first two FETP-Frontline cohorts were held from January to May, and from June to September 2017. Here, we report the results of a cross-sectional evaluation of these first two cohorts of FETP-Frontline in Guinea.

**Methods:**

The evaluation was conducted in April 2018 and consisted of interviews with graduates, their supervisors, and directors of nearby health facilities, as well as direct observation of data reports and surveillance tools at health facilities. Interviews and site visits were conducted using standardized questionnaires and checklists. Qualitative data were coded under common themes and analyzed using descriptive statistics.

**Results:**

The evaluation revealed a significant perception of improvement in all assessed skills by the graduates, as well as high levels of self-reported involvement in key activities related to data collection, analysis, and reporting. Supervisors highlighted improvements to systematic and quality case and summary reporting as key benefits of the FETP-Frontline program. At the health facility level, staff reported the training had resulted in improvements to information sharing and case notifications. Reported barriers included lack of transportation, available support personnel, and other resources. Graduates and supervisors both emphasized the importance of continued and additional training to solidify and retain skills.

**Conclusions:**

The evaluation demonstrated a strongly positive perceived benefit of the FETP-Frontline training on the professional activities of graduates as well as the overall surveillance system. However, efforts are needed to ensure greater gender equity and to recruit more junior trainee candidates for future cohorts. Moreover, although improvements to the surveillance system were observed concurrent with the completion of the two cohorts, the evaluation was not designed to directly measure impact on surveillance or response functions. Combined with the rapid implementation of FETP-Frontline around the world, this suggests an opportunity to develop standardized evaluation toolkits, which could incorporate metrics that would directly assess the impact of equitable field epidemiology workforce development on countries’ abilities to prevent, detect, and respond to public health threats.

**Supplementary Information:**

The online version contains supplementary material available at 10.1186/s12960-022-00729-w.

## Background

In 1998, the World Health Organization (WHO) Regional Office for Africa launched the Integrated Disease Surveillance and Response (IDSR) strategy to promote effective use of resources for disease surveillance and response using a district health approach. However, despite commitment from the various ministries of health, a 2008 assessment of the countries in the Economic Community of West Africa States (ECOWAS) found the successful implementation of a national IDSR strategy hindered by a lack of qualified public health personnel at all levels of the public health system [1]. A previous study also highlighted lack of workforce capacity as a major impediment to implementation of universal health coverage in the African region [2].

The 2014‒2016 Ebola virus disease (EVD) outbreak in West Africa originated in rural Guinea through a spillover of the virus into the human population [3]. EVD transmission then rapidly spread to Liberia and Sierra Leone, highlighting the three countries’ lack of public health infrastructure and system’s capacity to prevent, detect, and respond to emerging infectious disease threats [4] and meet WHO’s International Health Regulations (IHR) core capacity requirements for detecting, assessing, reporting and responding to potential public health emergencies of international concern. Establishing workforce capacity to prevent, detect, and respond to outbreaks and other potential public health emergencies is a critical component of the IHR.

Since 1980, the U.S. Centers for Disease Control and Prevention (CDC) has supported the establishment and implementation of Field Epidemiology Training Programs (FETPs) in over 80 countries. Modeled on CDC’s Epidemic Intelligence Service, the traditional FETP structure consists of 2 years applied, in-service training program which largely focuses on developing workforce capacity at the national level. The deficits identified during the West Africa EVD outbreak, however, emphasized the need to rapidly enhance the surveillance and outbreak investigation capacity of the public health workforce at the district level. In response, FETP-Frontline was developed in 2015 as a 3-month, accelerated training program in field epidemiology that specifically targets district level public health officials. The objective of FETP-Frontline is to improve the ability of the public health workforce to summarize and interpret surveillance data, prepare real-time surveillance reports, and participate in epidemiologic field investigations. Implementation of FETP-Frontline required extensive cooperation between the CDC and the Ministry of Health in each country to establish the need and feasibility of the program, conduct short implementation workshops for preliminary planning, and establish the priority of personnel to be trained. Mentors for the first cohorts were identified from neighboring countries if needed, and the countries were responsible for developing plans to ensure there would be adequate coverage of FETP graduates by the end of the program. To minimize health system disruptions, classroom activities were limited to 5-day sessions at the beginning, middle, and end of the program, sandwiched between extensive 5–6-week stints of field training. As with the Intermediate and Advanced FETP programs, FETP-Frontline uses an approach of 25% classroom activities and 75% on the job learning. By the end of 2016, 1,354 people from 24 countries had successfully completed the training [5]. In Guinea, the first two FETP-Frontline cohorts were held from January to May, and from June to September 2017, from which a total of 54 trainees graduated.

While there have been numerous evaluations both of national and regional full 2-year FETPs, as well as the impact of the FETP training model as a tool for global health workforce capacity building [6–10], to date there are few published accounts on the country-level benefits or impacts of FETP-Frontline [5, 11]. Here, we report the results of a cross-sectional evaluation of the first two cohorts of FETP-Frontline in Guinea. The objectives were to describe the characteristics of the graduates; how participation in FETP-Frontline was perceived by trainees and their supervisors; and how it improved the quality of their work. While impacts on the surveillance system were not directly assessed, the evaluation did also investigate the availability of key surveillance and reporting tools, associated with surveillance strengthening efforts..

## Methods

The evaluation consisted of interviews with graduates, their supervisors,^1^ and directors of health facilities that had served as field sites during the graduates’ training, as well as site visits for document checks of the graduates’ weekly reports, reporting tools, data summaries, and the surveillance tools at the same health facilities. The methods were based on FETP-Frontline evaluations conducted by CDC in Senegal and Côte d’Ivoire in 2016 and 2017, respectively, and were adapted to the Guinean context and implemented in the field by Research Triangle International (RTI), in collaboration with the African Field Epidemiology Network (AFENET) and CDC.

### Sample selection

**Fig. 1:**
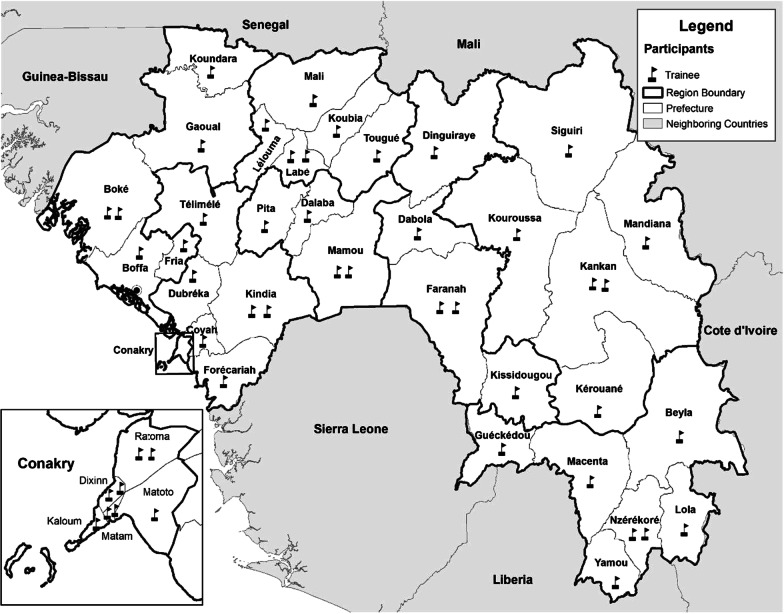
Map of districts staffed by Guinea FETP-Frontline graduates, Cohorts 1 and 2

After the completion of the second cohort, each of the country’s eight regions and 38 districts was staffed with at least one FETP-Frontline graduate (Fig. 1). The study population for the evaluation included all 54 graduates of the two cohorts, their current supervisors, and the director of one health facility, where each graduate conducted field exercises during their training. For the health facility interviews, the team reviewed the list of health facilities, where each graduate had conducted field exercises and selected one based on its proximity to the interviewee’s current work site. Proximity to the graduates’ current place of employment was the only criteria for health facility selection, due to transportation constraints preventing access to sites further away.

### Data collection

RTI staff with previous interviewing experience and familiarity with FETP trained four teams of two interviewers each, selected from two CDC staff, one RTI headquarter staff, and five local RTI field epidemiologists. All interviewers were fluent in French. The training included instructions on how to avoid response bias, probe for more detailed information, and listen actively by restating key points made by the interviewee.

RTI and AFENET revised questionnaires from previous evaluations to remove, edit, or add questions; changed some of the language to adapt it to the Guinean context (e.g., job titles); and reduced the number of open-ended questions. Two interview teams pilot-tested the participant questionnaire with graduates from the third cohort (not included in the evaluation) and recommended minor formatting changes to the interview tool.

The interviews were planned for April 10–20, 2018, corresponding to 11-month post-training for Cohort 1 graduates and 7-month post-training for Cohort 2. All interviews were conducted using the standardized questionnaires, covering pre-determined topic areas (Table 1). Full questionnaires are available as Additional file 1.

**Table 1:** Questionnaire topic areas for FETP-Frontline evaluation, Guinea

FETP graduate questionnaire	Demographics Self-assessed competency Data collection, analysis, and reporting practices Quality and content of surveillance reports (interviewer observation) Obstacles to implementation Recommendations
Supervisor questionnaire	Changes in work habits post-training Recommendations for additional training
Health facility staff questionnaire and observational checklist	Changes in surveillance practices post-field visit Obstacles to surveillance Reporting guidelines (case definitions, lists of immediate and mandatory notifiable diseases) posted (interviewer observed) Reports, data trends and archives, and rumor logs accessible (interviewer observed)

The interviews with the graduates were conducted in person, in private, at the graduates’ place of employment. Supervisors were interviewed in person where possible, or via telephone if a face to face meeting was not feasible. Interviews with the graduates lasted from 1 to 2 h, interviews with supervisors took up to 45 min, and interviews at the field visit health facilities took up to 30 min. At the facilities, interviewers conducted document checks and visual observations of the facilities, to assess availability of key guidance documents and other tools, and to validate information provided by the trainees.

### Team and data management

Interviewers worked in teams of two. Interviewers alternated between interviewing and note taking between each interview and attempted to transcribe verbatim the responses to the open-ended questions. Interviews were not recorded, as the team felt that recording might inhibit respondents, and especially graduates, from speaking freely. A data manager based in Conakry entered the complete responses into an Excel spreadsheet when the interview teams returned from the field. A second data manager then reviewed all the original questionnaires and compared them to the responses recorded in the database to confirm the responses in the database were complete and accurate. All team members involved in the data collection and management were fluent in French.

### Analysis

Descriptive statistics were summarized for each question using SAS® Enterprise Guide® (SAS Institute Inc., Cary, USA). All interviewee responses were included when calculating percentages unless otherwise noted in the table. After the data were entered into an Excel spreadsheet, a data manager identified common themes from participant answers to open-ended questions and developed a codebook listing all the possible codes for a specific question. Coding was done manually in the original French text so as not to lose nuances of the qualitative data. *P* values were calculated using *t* tests to compare differences in responses before and after participation in FETP-Frontline. The significance level was set at 0.05.

### Ethical statement

The study was determined to be exempt from IRB approval as primary data collection was conducted via interviews with individuals responding in their official capacity as trainees of a government program. The Ministry of Health provided permission to conduct the evaluation.

## Results

### Interview coverage

Between April 10–23, 2018, the team interviewed 50 (93%) of the 54 graduates. Of the four graduates not interviewed, three were away from their town of residence and could not reschedule their interviews despite multiple attempts, and one had retired 3 months before the study began. The team visited one health facility for each interviewed graduate (50 in total) to conduct site observations and interview staff and interviewed 37 (74%) of the supervisors. The remaining 13 supervisors were absent due to illnesses, trainings, or other reasons; the supervisors of the four graduates who were unable to be interviewed were not contacted for interviews.

### Demographic characteristics

Table 2 summarizes the demographic and professional characteristics of the interviewed graduates. Among those interviewed, 10 (20%) graduates were female and the mean age of the graduates was 48 years. The most frequently reported profession was physician (68%). On average, the graduates from the first cohort were older and had been on the job longer than the graduates from the second cohort. Job titles did not necessarily correlate to profession. For example, the health agents, the nurse, and the physicians all had the title Head of Disease Surveillance, which was the most commonly cited title (60%). The titles for those described as ‘other’ were Acting Regional Director of Health (*Direction Régionale de la Santé par intérim*), Chief of IHR Unit (*Chef d’unité Règlement Sanitaire International*) and Head of Community-Based Surveillance (*Chargé de Surveillance à Base Communautaire*). At the time of the interviews, all the graduates were in the same jobs they had when they participated in FETP-Frontline.

**Table 2:** Demographic characteristics of FETP-Frontline Guinea graduates interviewed (Cohorts 1 and 2)

Characteristics	Cohort 1, *N* = 24 *n* (%)	Cohort 2, *N* = 26 *n* (%)	Combined, *N* = 50 *n* (%)
Sex			
Female	6 (25%)	4 (15%)	10 (20%)
Male	18 (75%)	22 (85%)	40 (80%)
Age in years			
Mean	53 (NA)	44 (NA))	48 (NA)
Mean SD	8 (NA)	12 (NA)	11 (NA)
Median	52 (NA)	44 (NA)	50 (NA)
Median Range	32–62 (NA)	28–65 (NA)	28–65 (NA)
Profession			
Health Agent	3 (13%)	0 (0%)	3 (6%)
Epidemiologist	2 (8%)	1 (4%)	3 (6%)
Nurse	1 (4%)	0 (0%)	1 (2%)
Physician	11 (46%)	23 (88%)	34 (68%)
Public Health Specialist^a^	4 (17%)	2 (8%)	6 (12%)
Did not report	3 (13%)	0 (0%)	3 (6%)
Job title			
Deputy district head of disease surveillance and control	1 (4%)	10 (38%)	11 (22%)
Other	1 (4%)	2 (8%)	3 (6%)
Director of planning	3 (13%)	2 (8%)	5 (10%)
District head of disease surveillance and control	18 (75%)	12 (46%)	30 (60%)
Did not report	1 (4%)	0 (0%)	1 (2%)
Years at current position			
< 5	10 (42%)	17 (65%)	27 (54%)
5–10	5 (21%)	7 (27%)	12 (24%)
> 10	9 (38%)	2 (8%)	11 (22%)

N, total number; SD, standard deviation; NA , Not Applicable, in cases where it was not appropriate to calculate a percentage value for the data

^a^The “public health specialist” position is translated from *technician supérieur de santé publique*, which is a standardized position for nurses who have received additional training in public health, surveillance and epidemiology. In Guinea, the majority of public health functions are carried out by clinical professionals, such as doctors or nurses, in part reflecting the relative lack of separate public health career tracks and training within the educational system

### Self-assessment of skills pre- and post-training

The interview team asked graduates to rate their surveillance and data analysis skills on a one (novice) to five (expert) scale before and after the FETP-Frontline training. For both cohorts combined, there was a two-point increase in the mean of self-assessed level of skill in investigating outbreaks, verifying data quality, and summarizing data (standard deviation 0.72–0.75). The increase in skill level for using Excel and PowerPoint, and for describing a public health surveillance system was slightly less than two points (standard deviation 0.73–0.90). All differences were statistically significant (*p* ≤ 0.001) (Table 3).

**Table 3:** Self-assessed competency before and after participation in FETP-Frontline: both cohorts (*N* = 50)

Skill	Before Training	After Training	Difference	Percentage Improvement	*P* value
	Mean (SD)	Mean (SD)	Mean (SD)		
PowerPoint	2.47 (1.05)	4.03 (0.70)	1.56 (0.87)	63.2%	< 0.001
Excel	2.31 (0.99)	3.97 (0.66)	1.66 (0.90)	71.9%	< 0.001
Describing public health surveillance	2.43 (0.70)	4.23 (0.48)	1.79 (0.73)	73.7%	< 0.001
Investigating/Responding to outbreaks	2.20 (0.75)	4.20 (0.46)	2.00 (0.72)	90.9%	< 0.001
Summarizing data	2.15 (0.66)	4.24 (0.41)	2.09 (0.75)	97.2%	< 0.001
Verifying data quality	2.02 (0.75)	4.14 (0.57)	2.12 (0.75)	105.0%	< 0.001

The two cohorts did not have statistically significant differences in before and after scores, except for “verifying data quality”, for which Cohort 1 cited on average a slightly smaller difference in self-perceived improvement after the training (mean difference 2.02 versus 2.21; standard deviation 0.51 versus 0.91; *p* = 0.01).

### Data collection, analysis and reporting practices

Forty-seven graduates (94%) reported collecting data on notifiable diseases in 2 months prior to the interview (Table 4). Graduates reviewed original reports with reporting health facility staff to ensure the reports were complete, and then compared the data in the reports to data in the register. If there were any discrepancies between the register and the reports, graduates worked with health facility staff to resolve them and update the data. Graduates also performed other quality assurance activities including comparing data from the previous week, following up on missing or incomplete reports, and performing supervision visits.

**Table 4:** Survey of data collection, analysis, and reporting practices

	Yes	No
Data collection and quality assurance		
Have you collected data on notifiable diseases over the course of the last 2 months?	47 (94%)	3 (6%)
Have you conducted supervision visits at the sites that send you surveillance data over the course of the last 2 months?	45 (90%)	5 (10%)
Have you done a data quality audit over the course of the last 2 months?	34 (68%)	16 (32%)
Have you cleaned/validated surveillance data over the course of the last 2 months?	49 (98%)	1 (2%)
Have you participated in a case or outbreak investigation over the course of the last 2 months?	31 (62%)	19 (38%)
Analysis		
Have you recently tabulated and analyzed surveillance data?	48 (96%)	2 (4%)
Are your analyses of disease trends on display at your workplace?	41 (82%)	9 (18%)
Tools used for data analysis		
Excel Epi Info Manual analysis	49 (98%) 1 (2%) 2 (4%)	1 (2%) 49 (98%) 48 (96%)
Reporting		
Do you submit weekly surveillance reports to the next level in the disease surveillance system?	48 (96%)	2 (4%)
Format of reporting for those submitting weekly reports (*N* = 48):		
Email DHIS2	46 (97%) 33 (67%)	N/A

Thirty-one graduates (62%) reported they had participated in an investigation in 2 months prior to the interview. Forty-eight (96%) stated that in 2017, they had the opportunity to test their newly acquired skills during a measles outbreak. During this outbreak, 38 graduates (79%) investigated suspect cases, 21 (44%) investigated and reported outbreaks, 25 (52%) organized a response to the outbreak, 10 (21%) started a vaccination campaign, and nine (19%) provided community training.

Forty (80%) graduates provided data analysis results back to the health facilities that had sent the surveillance. They primarily share these analyses orally at routine weekly or monthly meetings held by the health facility. Only five (10%) graduates reported sharing the analyses by phone or email (data not shown). The primary reason the graduates did not provide analysis results to the health centers by email was because the health center staff lacked computer skills. Other barriers to sharing data analyses were lack of electricity or internet connection and lack of resources to produce hard copies of the analysis results.

**Fig. 2:**
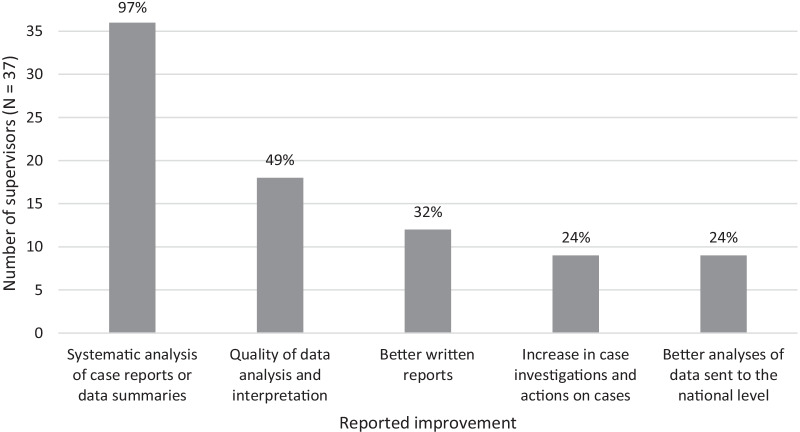
Summary of improvements reported by supervisors for FETP-Frontline program graduates

All but one supervisor cited the systematic analysis of case reports or data summaries as an improvement since the graduate completed training. Figure 2 summarizes the other improvements mentioned by the supervisors. Twenty-eight (76%) of supervisors indicated an improvement in the completeness and timeliness of the reports, as measured by reports that had no missing data and were submitted on time, whether weekly or monthly, compared to previous reports. Eighteen (49%) said the graduates’ reports included graphs and charts to summarize and interpret data, indicating improvement in quality and analysis of the data, and four (11%) said the data had documentation of validation, compared to previous reports. All but one of the supervisors (97%) reported that the graduates were routinely analyzing case reports and data summaries they received before submitting summary reports.

### Perceived benefit to the surveillance system

**Fig. 3:**
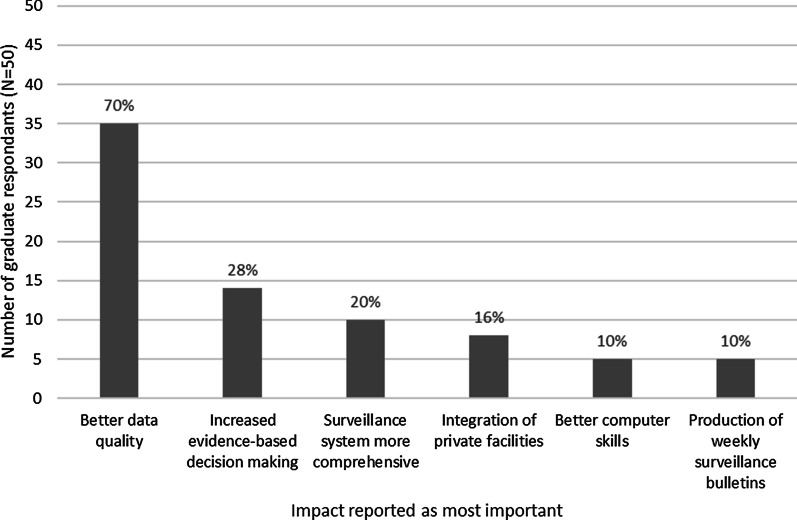
Graduate perceptions on the most important outcome of FETP-Frontline training on the surveillance system

Graduates reported that the most important outcome of their training was the improvement to data quality (Fig. 3). Twelve (30%) of graduates’ supervisors mentioned an improvement in the overall coordination and collaboration throughout the system, and 11 (30%) said the graduates were more motivated and engaged in surveillance activities.

In terms of perceived ability to implement measures to protect public health, 48 graduates (96%) said their analysis of the data enables them to follow the trends of reportable disease, identify outbreaks early, initiate investigations, and use surveillance data to make recommendations to improve public health or surveillance procedures. The graduates reported that based on their recommendations, there had been a vaccination campaign, integration of the private health clinics into the surveillance system, enhancements to the process for ensuring data quality, and additional funds or personnel. All supervisors reported that the graduates had worked to increase knowledge of case definitions in their districts, and 34 supervisors (92%) reported that the graduates had conducted a surveillance training in the district for community-level health facilities’ staff.

Forty-eight (96%) of the health facility staff interviewed said there were positive changes in surveillance and response activities since the graduate’s visit during their training. Twenty-nine interviewees (60%) reported that information sharing and case notifications had improved. Other improvements were reported in: (1) posting of case definitions in the facility; (2) availability of resources; (3) data quality; (4) use of computers; and (5) surveillance infrastructure. Indeed, the document checks and visual observations at the facilities revealed that case definitions were posted or easily accessible at 48 (96%) of the health facilities, though lists of immediately and mandatory reportable diseases were only observed in 27 (54%) of facilities. Graphs of data trends and rumor logs were only available in 11 (22%) and 8 (16%) of facilities, respectively.

### Barriers to implementation and suggestions for improvement

Thirty-six (72%) graduates reported encountering obstacles in carrying out surveillance activities after their training. The largest perceived obstacle was a lack of transportation, either because there was no vehicle or no fuel. Other problems included lack of personnel at the district health office, either because positions were not filled or because staff were away at trainings or other activities. Indecision at other levels of the surveillance system and a lack of information technology equipment were also cited as barriers. None of the graduates reported that either they or their colleagues were unfamiliar with surveillance concepts.

All graduates reported being satisfied with the training and with their current skills. They were concerned that if not provided with additional training, they would lose the momentum they had in the first year after completing the program. Several graduates asked for continuing education, and many of them were interested in being part of the FETP Intermediate cohort, a 9-month program also implemented in Guinea. Some graduates recommended enhancing the computer skills portion of the training, and many of them wanted continuing support and supervision from the program.

All supervisors suggested that the graduates would benefit from additional training on analysis software and project management, and that training like that provided by FETP-Frontline would also be of benefit to other employees of the health district.

## Discussion

The evaluation of the first two cohorts of Guinea’s FETP-Frontline program provides opportunities to make recommendations to improve the program as well as the evaluation process itself for future cohorts. While identified in the specific context of the Guinean program, some of these recommendations may also be applicable to other similar contexts, in West Africa and beyond.

Based on the demographic characteristics of the first two FETP-Frontline cohorts in Guinea, the evaluation revealed important future considerations for the recruitment of younger, and especially female, individuals to perform key surveillance functions in Guinea. Both initial cohorts were predominantly male (80%). One reason behind this disparity is that, because FETP-Frontline is intended to strengthen district surveillance systems, selection for the first two Frontline cohorts in Guinea focused on individuals serving as the District Head of Disease Surveillance and Control. Even though, according to the National Plan for Health Development (2015–2024) [12], women are equally represented in the overall health workforce (51% female to 49% male), the senior levels within the Ministry of Health, including in the districts, are primarily male. In this way, gender imbalance within FETP-Frontline trainees in Guinea is linked to underlying gender disparities within the Ministry of Health, which could be addressed through proactive policies to recruit women to serve as the heads of departments, including Departments of Disease Prevention and Control at the district level.

The relatively high mean age of the first cohort as compared to the second was part of a strategy to ensure the understanding and endorsement of the program from district health office leadership. In line with the strategy, subsequent cohorts, including the second cohort, targeted additional junior staff, responsible for the everyday surveillance and field epidemiology field work. Recruitment of junior staff is important to counterbalance the large proportion of the public health workforce in Guinea that will reach the mandatory age of retirement in the next 5 years. The Ministry of Health needs to prioritize provision of key skills to younger personnel, and particularly women, to galvanize the surveillance workforce and increase gender equity; these efforts may require close coordination with the higher education sector, to ensure a robust educational pipeline that specifically encourages graduates to pursue a career in the public sector. These challenges are not unique to Guinea; between 2013 and 2017, the regional 2-year West Africa Field Epidemiology and Laboratory Training Program trained 48 individuals from 11 countries, but only eight of the graduates were women (17%). The evaluation report noted that this disparity reflects underlying imbalances in the professionals from which trainees were recruited [13].

The evaluation revealed that FETP-Frontline graduates in Guinea were acutely aware of the skills they had gained from the training and were able to identify when and how to carry out local-level disease surveillance tasks, including not only collecting data and reporting it through identified channels, but also analyzing data and making recommendations for improvements to stakeholders. Findings indicate that graduates from both cohorts felt their skills had improved since participating in FETP-Frontline. Moreover, tangible outputs from FETP-Frontline graduates at the workplace were observed both by their supervisors and in the health facilities in which they served. For example, all but one of the health facilities had case definitions readily available. These findings mirror outcomes observed in other countries, as reported in the limited available published literature. In Côte d’Ivoire, for example, as in Guinea, the work of graduates of the Surveillance Training for Ebola Preparedness (STEP) program, which has similarities to FETP-Frontline in curriculum content, improved the understanding of case definitions and the quality of case investigations [14].

However, the health facility observations did reveal some gaps. Only just over half (54%) of the visited health facilities had lists of immediately or mandatory notifiable diseases visibly posted or readily available. It is possible that the absence of these guidelines from other facilities is due to the lists having been recently updated at the time of the evaluation, resulting from Guinea’s efforts to review, update, and enhance implementation of its national IDSR framework. However, in 2017, updated IDSR guidance, including lists of reportable diseases and their notification frequency, were distributed to all health facilities in the country. Given the newness of the guidelines, it is particularly important that they should be visibly displayed in all health facilities, to ensure staff are familiarized with the requirements. Similarly, very few (16%) of the facilities had a rumor log for suspected cases and outbreaks, and indeed most of the graduates seemed to be unfamiliar with the concept of a rumor log. Some even reported that it did not seem necessary when they had access to verifiable data to use for reporting. With event-based surveillance considered an important component for IHR compliance, and also incorporated into IDSR indicators, it will be important to better integrate these other aspects of surveillance within the FETP training structure. In that way, the planned expansion of FETP in Guinea and the subsequent the increase of FETP graduates working at all levels of the public health system will serve to address some of these observed deficiencies, as decision-makers have a better understanding of the tools required for a robust surveillance system.

A limitation of the evaluation was that it did not directly seek to measure impacts on the surveillance system. However, there is some evidence to suggest a positive benefit of the first two FETP-Frontline cohorts on the surveillance system in Guinea. Data collected during the training showed that the number of facilities integrated into the national surveillance system increased from 450 to 635. As is the case in many countries, the Ministry of Health in Guinea has limited control and oversight of private sector health facilities, and improving coordination with private for- and not-for-profit health providers is highlighted as a priority in the National Plan for Health Development 2015–2024 [12]. To this end, eight FETP-Frontline graduates in Guinea reported improved integration of private clinics into the national surveillance system as a product of the program. As an example, it was a private clinic, added to the surveillance system through the first FETP-Frontline cohort, which provided the MOH with sufficient data on the 2017 measles outbreak to allow for a formal request for WHO assistance, accelerating the response effort.

There are also data to support improved performance of the surveillance system, although these improvements cannot be directly attributed to the FETP-Frontline training. Timeliness of reporting, for example, increased from 68% around the start of the FETP-Frontline program to 98% by the time of the evaluation; the graduates themselves also perceived similar benefits. When the interview team asked graduates to identify the most important outcomes FETP-Frontline had had on the surveillance system, 70% reported that the quality, completeness, and timeliness of data had improved. This aligns with similar increases observed in other countries; for example, in Benin, average timeliness of surveillance increased almost 130% after the completion of the first cohort, from 37 to 85% in 12 weeks [5]. These data, coupled with the observations regarding the inclusion of private clinics, suggest that future evaluations could benefit from incorporating more direct measures of benefit, as well as impact, on the surveillance system, to further demonstrate the value provided by the FETP-Frontline training. Moreover, further investigations into the mechanisms and approaches used by the FETP-Frontline graduates to incentivize the participation of the private clinics could serve as useful models for other countries’ FETP and FETP-Frontline programs.

Another limitation of this evaluation of Guinea’s first two FETP-Frontline cohorts was that graduates were asked to score their pre- and post-training knowledge retrospectively, which could have resulted in recall bias. The extent of the recall bias could have been affected by the length of time after completion of the training that the questionnaires were completed, which differed between the two cohorts evaluated. Defining a set timeline for administering pre- and post-training questionnaires, and completing the post-training evaluation at a standard/routine interval after completion of the program, could reduce potential recall bias; indeed, CDC guidelines recommend conducting the evaluation no more than 6 months after the conclusion of the training, which was exceeded in the present evaluation. The possibility of desirability bias also cannot be discounted, whereby the respondents consciously or sub-consciously provided responses that would demonstrate the value of the program. The decision not to record the interviews might have helped mitigate some of this bias, as well as encourage the trainees to speak more freely, but reliance on note-taking by the interviewers might have resulted in some lost details or nuances in the recorded responses. The interview questions should also be reviewed by a survey design expert, to minimize ambiguity, leading questions, or other sources of inaccuracy, although the questionnaire was field-tested prior to deployment in the evaluation.

Given the successful implementation of FETP-Frontline across many countries since 2015 [5, 15], there may be an opportunity to develop a standardized monitoring and evaluation “toolkit”, consisting of suggested monitoring indicators aligned with IDSR, template evaluation questions, and other materials designed specifically for FETP-Frontline, which also incorporates standardized direct measures, such as the number of structures contributing to the surveillance system, as well as other indicators that could seek to examine the direct impacts on the FETP-Frontline program on the performance of the surveillance system. To our knowledge, examination of direct outcomes is a frequent omission in the evaluation of FETP and other related epidemiological workforce training programs, where the emphasis of evaluation is focused on pre- and post-training knowledge and competencies, participation in surveillance and response activities, and career progression of graduates [16–20]. To this end, a monitoring and evaluation toolkit could still have the flexibility to be customized to fit the specific needs and context of each country, but would standardize data collection between programs and allow for analysis of the benefits and impact of FETP-Frontline on a global, as well as national and regional, scale.

## Conclusions

The evaluation demonstrated a strongly positive perceived benefit of the FETP-Frontline training on the professional activities of graduates in support of surveillance and response functions, as well as the overall surveillance system. However, the demographic analysis of the first two cohorts revealed a substantial gender imbalance, and a tendency towards recruitment of more senior trainees approaching mandatory retirement age. Given how age and gender are aligned with seniority within the MoH, future recruitment efforts should seek to expand eligibility criteria to ensure more female and early-/mid-career trainees. Gaps were observed regarding the availability of notifiable disease lists, data analysis and trend reports, and especially rumor logs. Given the importance of these tools for accurate and timely reporting of emerging disease threats, in line with Guinea’s revised IDSR guidelines and IHR compliance requirements, future FETP-Frontline training should seek to improve availability and use of these instruments. Finally, although improvements to the surveillance system were observed concurrent with the completion of the two cohorts, the evaluation was not designed to directly measure impact on surveillance or response functions. Addressing this aspect of evaluation is critical for establishing an evidence-base to demonstrate the value-added of these epidemiological training programs, and in this way, be used to secure continued political and financial investment in workforce development to advance health security. Combined with the rapid implementation of FETP-Frontline around the world, this suggests an opportunity to develop standardized evaluation toolkits, which could incorporate metrics that would directly assess the benefit and impact of equitable field epidemiology workforce development on countries’ abilities to prevent, detect, and respond to public health threats, as well as alignment of desired competencies resulting from the training with other aspects of public health preparedness, response and resilience [21]. The recent establishment of new regional bodies, such as the Africa Centres for Disease Control and Prevention Institute for Workforce Development, present an important opportunity for leading and consolidating such efforts in the future [22].

## Supplementary Information

**Additional file 1**. FETP-Frontline Graduate Questionnaire.

## Data Availability

The data sets used and analyzed during the current study are available from the corresponding author on reasonable request.

## Footnotes

1 The supervisors were all either the director of the district health office (*Directeur de la Direction Préfectorale de Santé)* or the doctor in charge of disease control (*Médécin chargé des maladies*).
