# Supplementary material for: Evaluation of the first two Frontline cohorts of the field epidemiology training program in Guinea, West Africa
Source: Hum Resour Health. 2022 May 12;20:40. doi: 10.1186/s12960-022-00729-w (PMC9097411; doi:10.1186/s12960-022-00729-w)
Supplement: Supplementary file 1 — Additional file 1. FETP-Frontline Graduate Questionnaire. [file 12960_2022_729_MOESM1_ESM.docx]

FETP-Frontline Guinea:

Post-Training Cohort Evaluation

**Questionnaires**

**FETP-Frontline Graduate Questionnaire**

**Programme de Formation en Épidémiologie de Terrain Première ligne**

**(FETP-Première ligne)**

**Fiche de suivi des participants**

| **NOM & Prénom:** | **Âge :** |
| --- | --- |
| **Sexe: H / F** | **Tel:** |
| **Email:** | **Région:** |
| **Profession : Médecin**  **AS**  **Infirmier**  **Épidémiologiste**  **TSSE** | **Préfecture:** |
| **Titre/Fonction professionnel :**  **MCM**  **Charge d’étude**  **Assistant MCM**  **Autre__________________** | **Nombre d’années dans cette fonction :** |
| **Nom du superviseur :** | **Nom du Mentor :** |
| **Date de l’entretien :** |  |

**Introduction** (à lire au participant)

Merci d’avoir pris le temps de nous rencontrer aujourd’hui. Je souhaiterais vous poser quelques questions sur vos pratiques de surveillance depuis votre participation au Programme de formation en épidémiologie de terrain première ligne (FETP-F). Cet entretien ne prendra pas plus de 30 minutes. Vos commentaires permettront de continuer à améliorer le programme. Il n’y a pas de bonne ou mauvaise réponse. Nous souhaitons simplement savoir dans quelle mesure la formation a eu un impact sur votre travail. Vos réponses seront confidentielles, vous pouvez répondre ouvertement en toute confiance.

**Ceci est un instrument pédagogique, il n’y a ni de bonnes ni de mauvaises réponses.**

**Épidémiologie de terrain :**

| **Veuillez évaluer votre niveau de compétence AVANT et APRÈS la formation, sur une échelle de 1 à 5** | **AVANT LA FORMATION** | | | | | **APRÈS LA FORMATION** | | | | |
| --- | --- | --- | --- | --- | --- | --- | --- | --- | --- | --- |
| **1 = Aucune 2 = Novice 3 = Intermédiaire 4 = Avancé**  **5 = Expert** | **1** | **2** | **3** | **4** | **5** | **1** | **2** | **3** | **4** | **5** |
| Établir une liste linéaire des données collectées |  |  |  |  |  |  |  |  |  |  |
| Décrire en détail le cycle de surveillance épidémiologique |  |  |  |  |  |  |  |  |  |  |
| Utiliser la stratégie SIMR pour la surveillance des donnes de santé publique |  |  |  |  |  |  |  |  |  |  |
| Décrire pourquoi et comment se fait la collecte des données de santé publique |  |  |  |  |  |  |  |  |  |  |
| Décrire les étapes à suivre pour vérifier la qualité des données de santé publique |  |  |  |  |  |  |  |  |  |  |
| Utiliser les statistiques de base pour résumer les données de santé publique |  |  |  |  |  |  |  |  |  |  |
| Faire l’investigation d’un cas |  |  |  |  |  |  |  |  |  |  |
| Interpréter des données de surveillance |  |  |  |  |  |  |  |  |  |  |
| Décrire les étapes d’une investigation d’une flambée épidémique |  |  |  |  |  |  |  |  |  |  |
| Décrire les mesures de prévention et de contrôles adéquates |  |  |  |  |  |  |  |  |  |  |
| Faire le Suivi et l’évaluation d’un système de surveillance des données épidémiologiques |  |  |  |  |  |  |  |  |  |  |
| Faire un rapport hebdomadaire complet des données de surveillance épidémiologique |  |  |  |  |  |  |  |  |  |  |
| Rédiger un rapport complet d’investigation d’une flambée épidémique |  |  |  |  |  |  |  |  |  |  |

**L’Outil informatique**

| **Veuillez évaluer votre niveau de compétence AVANT et APRÈS la formation, sur une échelle de 1 à 5** | **AVANT LA FORMATION** | | | | | **APRÈS LA FORMATION** | | | | |
| --- | --- | --- | --- | --- | --- | --- | --- | --- | --- | --- |
| **1 = Aucune 2 = Novice 3 = Intermédiaire 4 = Avance**  **5 = Expert** | **1** | **2** | **3** | **4** | **5** | **1** | **2** | **3** | **4** | **5** |
| **PowerPoint** |  |  |  |  |  |  |  |  |  |  |
| Créer une présentation en utilisant un modèle existant |  |  |  |  |  |  |  |  |  |  |
| Effacer ou déplacer une diapositive dans une présentation |  |  |  |  |  |  |  |  |  |  |
| Formater une diapositive |  |  |  |  |  |  |  |  |  |  |
| Créer des graphiques |  |  |  |  |  |  |  |  |  |  |
| **EXCEL** |  |  |  |  |  |  |  |  |  |  |
| Créer une base de données |  |  |  |  |  |  |  |  |  |  |
| Filtrer et trier dans une base de données |  |  |  |  |  |  |  |  |  |  |
| Analyser une base de données |  |  |  |  |  |  |  |  |  |  |
| Utiliser Excel pour faire un histogramme et un graphique linéaire |  |  |  |  |  |  |  |  |  |  |
| Créer un tableau croise dynamique |  |  |  |  |  |  |  |  |  |  |

**PARTIE I: Activités réalisées au cours des deux derniers mois.**

| 1. Avez-vous collecté des données sur les maladies à déclaration obligatoire au cours des **deux derniers mois**? | ☐ **Oui** | ☐ **Non** |
| --- | --- | --- |
| 1. Avez-vous nettoyé/validé des données de surveillance au cours des **deux derniers mois**? | ☐ **Oui** | ☐ **Non** |
| 1. Avez-vous analysé les données de surveillance en élaborant des listes linéaires, des graphiques, des tableaux, etc. au cours des **deux derniers mois**? | ☐ **Oui** | ☐ **Non** |
| 1. Avez-vous effectué un audit de la qualité des données au cours des **deux derniers mois** ? | ☐ **Oui** | ☐ **Non** |
| 1. Est-ce qu’il y avait un cas confirmé d’une maladie à potentiel au cours des **deux derniers mois**? | ☐ **Oui** | ☐ **Non** |
| 1. Avez-vous développé des rapports de surveillance sur les maladies à potentiel épidémique (par exemple, la méningite, le choléra, etc.) au cours des **deux derniers mois**? | ☐ **Oui** | ☐ **Non** |
| 1. Avez-vous utilisé des données de surveillance pour formuler des propositions de recommandations aux niveaux régional et/ou national au cours des **deux derniers mois**? | ☐ **Oui** | ☐ **Non** |
| 1. Avez-vous participé à des investigations de cas ou de flambée épidémique au cours des **deux derniers mois**? | ☐ **Oui** | ☐ **Non** |
| 1. Avez-vous participé/effectué au moins une visite de supervision dans les sites qui vous envoient les données de surveillance au cours des **deux derniers mois** ? | ☐ **Oui** | ☐ **Non** |
| 1. Avez-vous formé quelqu’un sur l’analyse des données de surveillance au cours des **deux derniers mois**? | ☐ **Oui** | ☐ **Non** |
| 1. Avez-vous sensibilisé votre équipe-cadre de district sur votre travail pendant FETP ? | ☐ **Oui** | ☐ **Non** |

*Pour les enquêteurs : Pour les questions ci-dessous, veuillez poser la question, et voir le document en question. Cocher « oui » si le document est affiché ou facilement accessible, et « Non » dans le cas contraire.* ***S’il vous plait, prenez des photos de tous les éléments vus dans l’établissement de santé ou de poste.***

| 1. Dans votre poste de travail, y-a-t-il une **liste des maladies à déclaration obligatoire** affichée ou facilement disponible ? | ☐ **Oui** | ☐ **Non** |
| --- | --- | --- |
| 1. Dans votre poste de travail, y-a-t-il des **définitions de cas affichées** ou facilement accessibles ? | ☐ **Oui** | ☐ **Non** |
| 1. Dans votre poste de travail, utilisez-vous un journal de flambée et de rumeurs facilement disponible ? *(Cette question s’applique uniquement au niveau préfectoral)* | ☐ **Oui** | ☐ **Non** |
| 1. Dans votre poste de travail, y a-t-il des **analyses courantes des tendances** (par exemple, graphique linéaire, histogramme, etc.) affichées ou facilement disponibles pour les maladies prioritaires ? | ☐ **Oui** | ☐ **Non** |

**PARTIE II : Pour les questions suivantes, demandez au participant de répondre aussi honnêtement que possible, de détailler les réponses en vous donnant des exemples.**

1. Rapportage : Soumettez-vous des rapports hebdomadaires de surveillance ?  **Oui  Non**

Si oui, sous quelle forme est-elle soumise ?

1. Ecrit
2. Par appel téléphonique
3. Par SMS
4. Par e-mail (Base SAP)
5. DHIS2
6. Autre à préciser :

Si oui, montrez-nous le dernier rapport soumis

*A l’interviewer de vérifier le contenu*

*La promptitude et la complétude des rapports*  **Oui  Non**

*Cas*  **Oui  Non**

*Décès*  **Oui  Non**

1. Partagez-vous vos résultats avec les centres de santé à partir desquels vous avez reçu les données ?

**Oui  Non**

Sinon, pourquoi pas ?

1. Quelles mesures prenez-vous pour assurer la qualité des données ?
2. Suivi des rapports manquants/tardifs/incomplets
3. Comparaison avec les données des semaines précédentes
4. Vérification des données
5. Autre à préciser : ……………………………………………………………………………….
6. Comment les pratiques dont nous venons de discuter (et/ou la surveillance et les rapports en général), sont-elles différentes de celles que vous aviez l’habitude de suivre avant votre participation en FETP ?

**PARTIE III : Analyse des données, techniques et outils**

1. Analysez-vous régulièrement les données de la surveillance épidémiologique ?  **Oui  Non**

**Si non, passez directement à QUESTION 21.**

Si oui, quels outils utilisez-vous pour représenter les données ?

|  | Diagrammes à barres | ☐ **Oui** | ☐ **Non** |
| --- | --- | --- | --- |
|  | Des histogrammes | ☐ **Oui** | ☐ **Non** |
|  | Des graphiques linéaires | ☐ **Oui** | ☐ **Non** |
|  | Des cartes | ☐ **Oui** | ☐ **Non** |
|  | Autre | ☐ **Oui** | ☐ **Non** |
|  | Précisez « autre » : |  | |

1. Dans votre dernier rapport, quels outils d’analyse avez-vous utilisés pour analyser les données?

|  | Manuellement | ☐ **Oui** | ☐ **Non** |
| --- | --- | --- | --- |
|  | MS Excel | ☐ **Oui** | ☐ **Non** |
|  | EPI-Info | ☐ **Oui** | ☐ **Non** |
|  | EPI-Data | ☐ **Oui** | ☐ **Non** |
|  | Autre | ☐ **Oui** | ☐ **Non** |
|  | Précisez « autre » : |  | |

Après votre formation FETP, est-ce que votre analyse des données de surveillance vous a permis de suivre la tendances des maladies sous surveillance, l’identification des flambées épidémiques à partir des seuils ou a motivé d’autres investigations ? **Oui  Non**

Si oui, citer les actions prises

Après votre formation en FETP, avez-vous utilisé les données de surveillance pour formuler des recommandations pour l’amélioration de la santé publique ou le processus de surveillance ?

**Oui  Non**

(a) Si Oui, citer quelques recommandations prises sur base de ces informations

(b) Y-a-t-il eut des actions prises suite à vos recommandations ?  **Oui  Non**

(c) Si Oui, citer des actions réalisées suites aux recommandations effectuées

**PARTIE IV :**

1. Y a-t-il eu des obstacles à la poursuite de vos travaux de surveillance après la formation FETP-Première ligne ?  **Oui  Non**

Si oui, lesquels ?

1. Absence de transport
2. Absence au travail due à des formations
3. Manque de connaissance sur un sujet
4. Autre à préciser :
5. Y a-t-il eu des activités de surveillance que vous vouliez faire, mais que vous ne pouviez pas faire à cause d’un obstacle ?  **Oui  Non**

Si oui, pouvez-vous nous donner quelques exemples d’activités et d’obstacles qui l’ont empêché ?

1. Quel est l’impact le plus important que vous avez observé sur le système de surveillance après la formation ?

1. Avez-vous des questions à nous poser en rapport avec l’interview ?

**PARTIE V :** Demandez au participant de vous montrer le dernier rapport de surveillance qu’il/elle a préparé. Examinez le rapport et indiquez si le participant a utilisé les techniques et les outils d’analyse décrite pendant cet entretien. Et marquez quel type de rapport (hebdomadaire, etc.).

1. *Pour l’enquêteur :*

*Est-ce que c’est un rapport hebdomadaire, etc. ?*

*Est-ce que le participant a :*

*(a) fait des graphiques ?*  ☐ **Oui** ☐ **Non**

*(b) tracé des courbes ?* ☐ **Oui** ☐ **Non**

*(c) fait des tableaux ?* ☐ **Oui** ☐ **Non**

*(d) fait quelque chose d’autre (à préciser)?*

***Prenez des photos si possibles.***

1. Pouvez-vous nous montrer les 12 derniers rapports hebdomadaires :

a) le nombre de sites qui ont soumis leurs données de surveillance à temps au cours des 12 derniers semaines épidémiologiques :

b) Le nombre de sites qui ont soumis leurs données de surveillance complètes au cours des 12 derniers semaines épidémiologique :

c) Le nombre de sites dans votre district:

1. Quelles sont vos suggestions et recommandations pour l’amélioration du Programme FETP-Première ligne ?

**page de signature**

| **NOM & Prénom Interviewer:** | **NOM & Prénom Interviewé:** |
| --- | --- |
| **Signature Interviewer:** | **Signature Interviewé:** |
| **Date :** | **Date :** |

**FETP–Frontline Supervisor Questionnaire**

**Programme de Formation en Épidémiologie de Terrain Première ligne**

**(FETP-Première ligne)**

**Fiche de suivi des participants des Cohortes 1 & 2**

**Évaluation des performances/Entretien : Superviseur**

| **Nom & Prénom :** | |
| --- | --- |
| **Sexe :** | **Tel :** |
| **Email:** | **Région :** |
| **Profession** | **District :** |
| **Titre professionnel :** | **Nombre d’année dans cette fonction :** |
| **Date de l’entretien :** |  |

**Introduction** (à lire au supérieur hiérarchique)

Merci d’avoir pris le temps de nous rencontrer aujourd’hui. Je souhaiterais vous poser quelques questions sur les pratiques de surveillance de votre employé depuis sa participation au Programme de formation en épidémiologie de terrain première ligne (FETP-F). Cet entretien ne prendra que 15 à 20 minutes. Vos commentaires permettront de continuer à améliorer le programme. Il n’y a pas de bonne ou de mauvaise réponse. Nous souhaitons simplement savoir dans quelle mesure la formation a eu un impact sur le travail des participants. Vos réponses seront confidentielles, vous pouvez répondre ouvertement en toute confiance.

**Quelle est votre relation professionnelle avec le participant ?**

| **Participant FETP Première ligne** | **Relation avec le participant** |
| --- | --- |
| **MCM** |  |
| **Suppléant MCM** |  |
| **Chargé d’étude** |  |
| **Autre** |  |

1. **Quels changements avez-vous observés dans le travail du participant depuis sa participation à la formation ? Veuillez décrire ces changements et donner des exemples.**

| **Participant FETP Première ligne** | **Changements observés** |
| --- | --- |
| **MCM** |  |
| **Suppléant MCM** |  |
| **Chargé d’étude** |  |
| **Autre** |  |

1. **Le participant a-t-il travaillé pour accroître les connaissances sur les définitions de cas des maladies et événements sous surveillance dans votre district ?**

| **Participant FETP**  **Première ligne** | **Connaissances**  **sur les définitions de cas** | **Le cas échéant, veuillez fournir des exemples** |
| --- | --- | --- |
| **MCM** | ☐ **Oui** ☐ **Non** |  |
| **Suppléant MCM** | ☐ **Oui** ☐ **Non** |  |
| **Chargé d’étude** | ☐ **Oui** ☐ **Non** |  |
| **Autre** | ☐ **Oui** ☐ **Non** |  |

1. **Quels changements avez-vous remarqués dans le contenu et la qualité des rapports de surveillance ? Veuillez décrire ces changements et donner des exemples**

| **Participant FETP**  **Première ligne** | **Contenu et qualité des rapports de surveillance : changements et exemples** |
| --- | --- |
| **MCM** |  |
| **Suppléant MCM** |  |
| **Chargé d’étude** |  |
| **Autre** |  |

1. **Le participant analyse-t-il systématiquement les rapports sur les cas et/ou les synthèses de surveillance ? Cela a-t-il entraîné des changements ? Si possible, donnez un exemple.**

| **Participant FETP-F** | **Analyse temps-Lieu-Personnes des données de surveillance** | **Si OUI : changement observé et exemples** |
| --- | --- | --- |
| **MCM** | ☐ **Oui** ☐ **Non** |  |
| **Suppléant MCM** | ☐ **Oui** ☐ **Non** |  |
| **Chargé d’étude** | ☐ **Oui** ☐ **Non** |  |
| **Autre** | ☐ **Oui** ☐ **Non** |  |

1. **Le participant a-t-il conduit une formation sur la surveillance dans le district après le programme FETP-F ?**

| **Formation sur la surveillance épidémiologique** | **Si OUI, Qui a-t-il formé ?** | **Si OUI, quels changements cette formation a-t-elle entraînés ? Soyez précis.** |
| --- | --- | --- |
| **MCM** | ☐ **Oui** ☐ **No** |  |
| **Suppléant MCM** | ☐ **Oui** ☐ **Non** |  |
| **Chargée d’étude** | ☐ **Oui** ☐ **Non** |  |
| **Autre** | ☐ **Oui** ☐ **Non** |  |

**Avez-vous participé à la formation ?** ☐ **Oui** ☐ **Non**

1. **Veuillez décrire les autres changements que vous avez observés, aussi bien positifs que négatifs, suite à sa participation au programme FETP Première ligne.**

| **Formation sur la surveillance épidémiologique** | **Changements observés** |
| --- | --- |
| **MCM** |  |
| **Suppléant MCM** |  |
| **Chargé d’étude** |  |
| **Autre** |  |

1. **Recommanderiez-vous que d'autres employés reçoivent une formation similaire ?**

**☐** **Oui** ☐ **Non**

1. **Quelles autres compétences/formations pourraient aider les participants à effectuer leur travail ?**

| **Participants** | **Autres formations nécessaires** |
| --- | --- |
| **MCM** |  |
| **Suppléant MCM** |  |
| **Chargé d’étude** |  |
| **Autre** |  |

1. **Quelles sont vos besoins en matière de formation pour mieux superviser les participants en ce qui concerne la prévention, la détection et la riposte face aux urgences de santé publique.** Vous trouverez ci-dessous certaines propositions, veuillez nous faire des suggestions. Cette formation pourrait inclure les questions portant sur :

Concepts épidémiologiques de base

RSI et GSHA

Concept «One Health»

SIMR et surveillance à base communautaire

Maladies évitables par la vaccination

Leadership

Management

Gestion des crises et des risques associés aux urgences de santé publique

Prise de décisions fondée sur des données probantes en santé publique

Évaluation

Autres :

1. **Avez-vous d'autres commentaires ou recommandations pour FETP-Première ligne?**

**page de signature**

| **NOM & Prénom Interviewer:** | **NOM & Prénom Interviewé:** |
| --- | --- |
| **Signature Interviewer:** | **Signature Interviewé:** |
| **Date :** | **Date :** |

**FETP-Frontline Health Facility Field Site Questionnaire**

**Programme de Formation en Épidémiologie de Terrain Première ligne**

**(FETP-Première ligne)**

**Les visites sur le terrain** (Formation sanitaire suivie)

Nom de participant :

Nom de District :

Nom de la formation sanitaire:

Date :

Questions pour les agents à la formation sanitaire

1. Est-ce que vous avez noté des changements dans la pratique de la surveillance et/ou la riposte après sa visite et sa formation? ☐ **Oui** ☐ **Non**

Si oui, décrivez :

1. Quels sont les obstacles dans votre travail qui vous empêchent de bien faire la surveillance?

Pour les éléments suivants, vérifiez s’ils sont affiché ou disponible facilement. **Cochez si c’est *facilement* disponible.**

NB : prenez les photos si possibles.

| **Affiché** | | **Facilement Disponible** | |
| --- | --- | --- | --- |
| Les graphiques |  | Registre des rumeurs |  |
| La liste des maladies sous surveillance a déclaration immédiate obligatoire |  | Archivage des données |  |
| La liste des maladies sous surveillance a déclaration obligatoire |  | Les définitions de cas |  |
| Les définitions de cas |  | Les rapports de surveillance |  |

**page de signature**

| **NOM & Prénom Interviewer:** | **NOM & Prénom Interviewé:** |
| --- | --- |
| **Signature Interviewer:** | **Signature Interviewé:** |
| **Date :** | **Date :** |
